# Phylogenetic Codivergence Supports Coevolution of Mimetic *Heliconius* Butterflies

**DOI:** 10.1371/journal.pone.0036464

**Published:** 2012-05-07

**Authors:** Jennifer Hoyal Cuthill, Michael Charleston

**Affiliations:** School of Information Technologies, University of Sydney, Sydney, New South Wales, Australia; Field Museum of Natural History, United States of America

## Abstract

The unpalatable and warning-patterned butterflies *Heliconius erato* and *Heliconius melpomene* provide the best studied example of mutualistic Müllerian mimicry, thought–but rarely demonstrated–to promote coevolution. Some of the strongest available evidence for coevolution comes from phylogenetic codivergence, the parallel divergence of ecologically associated lineages. Early evolutionary reconstructions suggested codivergence between mimetic populations of *H. erato* and *H. melpomene*, and this was initially hailed as one of the most striking known cases of coevolution. However, subsequent molecular phylogenetic analyses found discrepancies in phylogenetic branching patterns and timing (topological and temporal incongruence) that argued against codivergence. We present the first explicit cophylogenetic test of codivergence between mimetic populations of *H. erato* and *H. melpomene*, and re-examine the timing of these radiations. We find statistically significant topological congruence between multilocus coalescent population phylogenies of *H. erato* and *H. melpomene*. Cophylogenetic historical reconstructions support repeated codivergence of mimetic populations, from the base of the sampled radiations. Pairwise distance correlation tests, based on our coalescent analyses plus recently published AFLP and wing colour pattern gene data, also suggest that the phylogenies of *H. erato* and *H. melpomene* show significant topological congruence. Divergence time estimates, based on a Bayesian coalescent model, suggest that the evolutionary radiations of *H. erato* and *H. melpomene* occurred over the same time period, and are compatible with a series of temporally congruent codivergence events. Our results suggest that differences in within-species genetic divergence are the result of a greater overall effective population size for *H. erato* relative to *H. melpomene* and do not imply incongruence in the timing of their phylogenetic radiations. Repeated codivergence between Müllerian co-mimics, predicted to exert mutual selection pressures, strongly suggests coevolution. Our results therefore support a history of reciprocal coevolution between Müllerian co-mimics characterised by phylogenetic codivergence and parallel phenotypic change.

## Introduction

The Neotropical butterfly genus *Heliconius* (Nymphalidae, Heliconiinae) is highly diverse, with 39 species [1], [2], many of which can be subdivided into multiple wing pattern morphs, or races [3]. These unpalatable butterflies [4], [5] have diversified to form regional Müllerian [6] mimicry complexes [7], each involving multiple species with a convergently evolved [8] predator-warning pattern [5], [9]. For almost 150 years, biologists have debated whether the remarkable adaptive radiation of the *Heliconius* was driven by reciprocal ecological associations, a process now known as coevolution [10]. Unpalatable Müllerian [6] co-mimics share the cost of educating inexperienced predators [5] (unlike palatable Batesian [7] mimics, which may “parasitize” their unpalatable models [11]). According to Müller's original model [6], two unpalatable co-mimics will both gain in fitness by their resemblance, though the ratio of these fitness gains will be proportionate to the square of the ratio of their population sizes. Where population sizes differ, this predicts greater fitness benefits for the rarer population because the more abundant co-mimic is expected to lose a greater number of individuals to encounters with inexperienced predators [11]. It has been suggested that mimicry (and, particularly, mutualistic Müllerian mimicry [11]) may provide some of the most favourable conditions for coevolution, which has been defined (in the strict sense) as reciprocal evolutionary change [12] under mutualistic or competitive selection [13]. Therefore, mimetic wing pattern evolution among *Heliconius* butterflies may provide key evidence regarding the importance of coevolution in adaptive radiation [10], [14].

**Figure 1:**
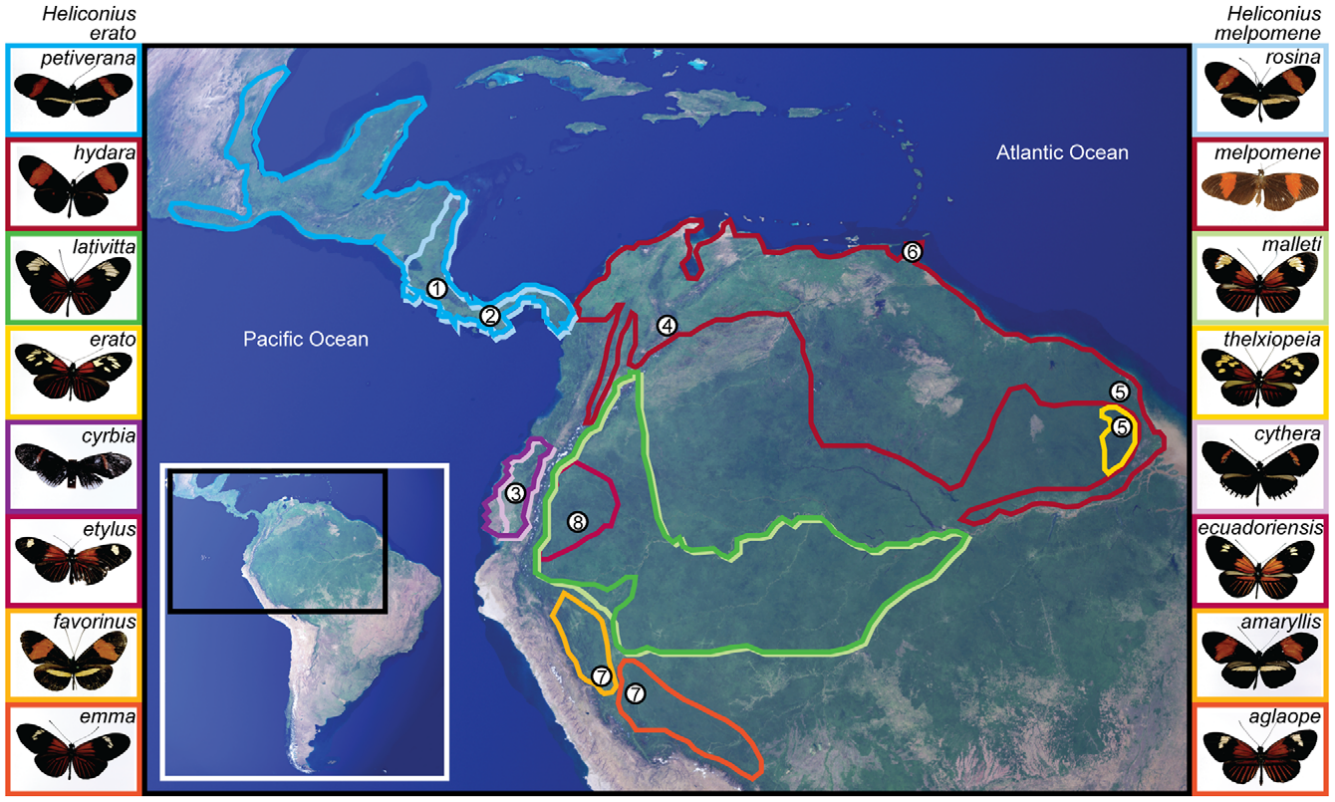
Wing patterns morphs and geographic distributions of the Müllerian co-mimics *H. erato* and *H. melpomene*. Mimetic morphs [3], [15], [30] (aligned rows), from *H. erato* (left) and *H. melpomene* (right), included in this study (photographs show the type specimens). Coloured boundaries on a satellite image of Central and South America indicate the geographic range of each morph [3]. Numbers indicate countries where morphs were sampled (by [29], [30]), West of the Andes: 1 Costa Rica, 2 Panama, 3 West Ecuador; East of the Andes: 4 Colombia, 5 French Guiana, 6 Trinidad, 7 Peru and 8 East Ecuador.

In particular, the parallel wing pattern radiations of *H. erato* and *H. melpomene* have been a primary case study in the debate over coevolution between Müllerian co-mimics [3], [14], [15], [16]. Both *H. erato* and *H. melpomene* are unpalatable and have wing colour patterns that deter potential predators [9], [17], [18]. Across the Americas, each of these two species exhibits approximately 30 warning pattern morphs [3]. With few exceptions, the wing patterns of *H. melpomene* and *H. erato* match in every region where they co-occur [3] (Figure 1), suggesting that they are Müllerian co-mimics [3], [19], [14]. The two species are reciprocally monophyletic [20] and do not hybridise [21]. Therefore mimicry between them has involved convergence at the genetic and phenotypic level [8], [22]. Purifying selection against intra-species hybrids with unusual wing patterns [5] acts with assortative mating [23] to generate partial reproductive isolation between the parapatric morphs [15], potentially showing speciation in action [3], [7], [24].

Codivergence is the parallel divergence of ecologically associated lineages within two distinct phylogenies [25], and is one predicted outcome of coevolution [11], [26]. Codivergence may not, in itself, prove coevolution in the strict sense [14]. However, codivergence can be considered some of the strongest available evidence for coevolution [14], [26], since, as Page [25] states, “it is difficult to imagine that [codivergence] can occur without at least some degree of coevolution”. Topological and temporal congruence (similarity of branching pattern and timing, respectively) between the phylogenies of *H. erato* and *H. melpomene*, compatible with a history of codivergence, would therefore support their coevolution [3], [14], [15], [27] (in reference to their Pleistocene refugium hypothesis, Brown et al. [19] and Sheppard et al. [3] suggested that coevolution between *H. erato* and *H. melpomene* may have been aided by population isolation, but see [28] for a critical review). In contrast, a lack of topological or temporal congruence would suggest that coevolution did not occur (as previously suggested [15], [29], [30]).

Despite considerable discussion of phylogenetic branching patterns [3], [15], [30] and timing [15], [29], [30], and an early biogeographic character-based analysis [15], there has been no previous test of codivergence between the mimetic populations of *H. erato* and *H. melpomene* using methods from cophylogenetic analysis (reviewed in [25]), which were developed specifically for this purpose. Cophylogenetic analysis tests for topological congruence between a pair of phylogenies that represent associated entities (such as genes and species, parasites and their hosts, populations and biogeographic regions [25], or mimics and models [31]) to determine whether there is statistically significant evidence for codivergence between these associated phylogenies (as described below). We present the first explicit cophylogenetic test for codivergence between mimetic populations of *H. erato* and *H. melpomene*, using multilocus coalescent [32], [33] phylogenies. We then re-examine divergence times based on the Bayesian multilocus coalescence model [33], using an established [34] – and recently corroborated [35] – fossil-calibrated molecular clock, as well as a root-node calibration based on the most comprehensive published butterfly phylogeny [36].

One of the greatest challenges in reconstructing phylogenetic branching patterns and divergence times for recent radiations with low sequence divergence, such as that of the *Heliconius*, is the incomplete sorting of ancestral polymorphisms among divergent populations [37]. Incomplete lineage sorting can cause individual gene trees to conflict with each other and with the true population tree. A related problem is that individuals sampled from divergent lineages may not form reciprocally monophyletic clades on an individual gene tree, or on a population tree built using gene sequence concatenation or gene tree consensus methods [38]. Coalescent methods are designed to take individual gene histories into account by modelling the processes of mutation and inheritance, specifically, the coalescence of sampled genes, back through a gene tree, to their most recent common ancestor (reviewed by Rosenberg and Nordborg [37]). Coalescent phylogenetic methods reconstruct the relationships between divergent populations, that are partially or completely genetically-isolated [39], [40], by optimally reconciling the histories of multiple gene loci within one population-level tree [32], [33]. Coalescent methods have rarely been applied to heliconian population genetics [29], and have not previously been used to reconstruct the phylogenies of *H. erato* and *H melpomene*.

In this study, we use coalescent [33] and character support [41] methods to delimit monophyletic populations of *H. erato* and *H. melpomene*, among individuals sampled at the level of country, biogeographic region, and morph (Table S1). Phylogenetic relationships [32], [33] and divergence times [33] for these populations are reconstructed using Bayesian [33] and parsimony based (Minimise Deep Coalescence, MDC [32]) coalescent methods. These phylogenies provide the basis for cophylogenetic tests of topological congruence, conducted across the set of corresponding phylogenetic estimates returned by the coalescent analyses. Estimated branching patterns, cophylogenetic histories, and divergence times are presented and discussed, based primarily on phylogenies of the country level mimetic populations, which received the highest support for monophyly from the character based [41] and Bayesian coalescent [33] analyses. To ensure that the conclusions are not exclusive to our coalescent population phylogenies, we also conduct cophylogenetic analyses on phylogenies reconstructed from recently published amplified fragment length polymorphism (AFLP) [30] and colour pattern gene [22] data.

## Materials and Methods

### Molecular data, phylogenetic and population genetic analysis

The primary dataset of 290 multilocus DNA sequences (Table S1) included eight pairs of mimetic wing pattern morphs from *H. erato* and *H. melpomene* (as well as the morphs *H. erato chestertonii* and *H. melpomene plesseni*, whose co-mimics were not included in this study), sampling populations from the major Neotropical biogeographic regions (East and West of the Andes) and seven countries [29], [30] (Figure 1). Twenty-two related species [20], [29], [30], [42] were also included, in order to estimate positions and divergence times, for the population radiations of *H. erato* and *H. melpomene*, within the wider radiation of genus *Heliconius*. These species, and morphs of *H. erato* and *H. melpomene*, were selected to maximise coverage of the four included gene loci.

**Table 1:** Significance of topological congruence between *H. erato* and *H. melpomene* phylogenies.

Phylogenies	Minimum Cost (*p* [95% max])		Distance Correlation (*p* [95% max])	
	Random Associations	Random Mimic Tree	Root of Mimic (*H. melpomene*)	Root of Model (*H. erato*)
Separate MDC countries 1	0 [0.0024]	0 [0.0024]	0.003 [0.0074]	0.001 [0.0042]
Separate MDC countries 2	0 [0.0024]	0 [0.0024]	0.002 [0.0059]	0 [0.0024]
Separate MDC regions 1	0.200 [0.2254]	0.181 [0.2054]	0 [0.0024]	0 [0.0024]
Separate MDC morphs 1	0.025 [0.0354]	0.016 [0.0245]	0.004 [0.0089]	0.001 [0.0042]
combined MDC countries 1	0.005 [0.0103]	0.004 [0.0089]	0.001 [0.0042]	0 [0.0024]
combined MDC countries 2	0.003 [0.0074]	0 [0.0024]	0 [0.0024]	0 [0.0024]
combined MDC countries 3	0.005 [0.0103]	0 [0.0024]	0.002 [0.0059]	0.004 [0.0089]
combined MDC countries 4	0 [0.0024]	0 [0.0024]	0.001 [0.0042]	0.001 [0.0042]
combined MDC regions 1	0.225 [0.2514]	0.212 [0.2379]	0.017 [0.0257]	0.015 [0.0233]
combined MDC morphs 1	0.547 [0.5784]	0.529 [0.5605]	0.054 [0.0686]	0.086 [0.1040]
combined *BEAST countries	0.029 [0.0401]	0.029 [0.0401]	0.088 [0.1061]	0.077 [0.0941]
combined *BEAST morphs	0.003 [0.0074]	0.019 [0.0282]	0.007 [0.0130]	0.005 [0.0103]
Quek et al., 2010 AFLP	N/A		0 [0.0024]	0 [0.0024]
Hines et al. 2011, 10-genes	N/A		0 [0.0001]	0 [0.0001]
Hines et al. 2011 colour pattern genes	N/A		0 [0.0001]	0 [0.0001]

Phylogenies were estimated separately for the clades of *H. erato* and *H. melpomene* and in a combined analysis of the *Heliconius*. The significance of the cophylogeny mapping analyses was estimated based on minimising total reconstruction costs with Jane 3, either by randomising the leaf associations (column 2) or by randomizing the *H. melpomene* phylogeny (column 3). The significance of the pairwise distance correlation was calculated, at the root of the *H. melpomene* phylogeny (column 4) and at the root of the *H. erato* phylogeny (column 5), using TreeMap 3. Each *p*-value was estimated with 1000 Monte-Carlo replicates, and the 95% confidence upper bound was calculated for each using Wilson's score interval for binomial proportions [68].

This primary dataset comprised 3,533 base pairs sampled from four gene loci, namely the mitochondrial loci cytochrome c oxidase COI and COII, and the nuclear loci mannose 6-phosphate isomerase (Mpi) and triose-phosphate isomerase (Tpi). DNA sequences were downloaded from GenBank (accession numbers and source studies Table S1). Sequences of each gene locus were aligned using MUSCLE [43]. Alignments for individual gene loci were used in Maximum Likelihood (ML) phylogenetic analyses, implemented in Treefinder [44], to produce individual gene trees as input for the MDC [32] coalescent phylogenetic analyses (described below). The ML phylogenetic analyses were conducted using the best-fit substitution model for each gene, selected by jModelTest 0.1.1 [45] under the Akaike information criterion (AIC) [46]. Sequences for the four included gene loci were also concatenated to produce a multilocus data matrix (Table S3).

Population genetic statistics were estimated based on the multilocus data matrix, using the program SITES [47]. These statistics included the average pairwise genetic divergence within a population and an effective population size parameter, θ, estimated as the product of effective population size and mutation rate (θ = 4 N_e_μ where N_e_  =  effective population size and μ is the neutral mutation rate [48], [49]). Two tests of population monophyly were performed, on the multilocus data matrix, for specimens of *H. erato* and *H. melpomene* grouped at the level of country, biogeographic region, morph or species. First, monophyly was assessed with Shimodaira-Hasegawa (SH) tests [41] on monophyly-constrained ML trees, using Treefinder. This test compared the support for each population level using the AIC, which evaluates the fit of a statistical model to the data against the number of parameters imposed by that model – in this case, the number of constraints required for monophyly at the given population level. Since the AIC is a measure of information loss, the preferred phylogenetic hypothesis will be the one with the lowest AIC value. Incongruence length difference (ILD) tests, conducted using PAUP* 4.0b10 [50], indicated significant incongruence between the nuclear loci for both *H. erato* and *H. melpomene* (*p* = 0.01 in each case), so the nucleotide substitution model was partitioned by gene locus (COI, GTR+I+G; COII, HKY+I+G; Mpi, GTR+G; Tpi, HKY+G). The second test of population monophyly was based on an explicit Bayesian multilocus coalescent model [33] and conducted using Bayesian phylogenies reconstructed for each geographical sampling level (further details below).

Coalescent population phylogenies were reconstructed by minimising the number of deep gene coalescences [32] in Mesquite [51] (conducted separately for the clades of *H. erato* and *H. melpomene* and for the combined taxon set), and using a Bayesian multi-population coalescent model in *BEAST [33], part of the BEAST 1.6.1 package [52] (for the combined taxon set). Such methods, which are based on an explicit model of gene lineage coalescence, have been found to accurately reconstruct population level phylogenies and are robust to low levels of gene flow [39], [40]. MDC [32], [51] phylogenies were each reconstructed using a heuristic, population level tree search, which incorporated the branch lengths of the four gene trees, did not auto-resolve gene tree polytomies, used subtree pruning and regraft branch-swapping, and stored up to 100 equally good trees at each search step. Bayesian coalescent analyses were based on partitioned nucleotide substitution models, selected under the Bayesian information criterion (COI, HKY+I+G; COII, HKY+I+G; Mpi, HKY+G; Tpi, HKY+G). The focal *BEAST analyses were run using a relaxed, uncorrelated log-normal molecular clock (selected based on Bayes factor comparisons against an, otherwise identical, analysis run with a strict molecular clock), allowing the mutation rate to vary within the phylogeny [52]. A Yule prior was specified for the branching process of the population tree. Since the two mitochondrial gene loci (COI and COII) are non-recombining, a linked tree was specified for these loci in the *BEAST analyses [52]. Each *BEAST analysis was run with a Markov chain Monte Carlo (MCMC) chain length of 10^8^ steps, parameter sampling every 10^4^ steps, and a conservative burn-in of 25%. Effective sample size (ESS) values, for the posterior distribution of each parameter, were assessed to check chain convergence in each *BEAST run. Output from *BEAST was analysed using Tracer [52] and visualised using FigTree 1.3.1 (A. Rambaut, http://tree.bio.ed.ac.uk/software/figtree/). The MDC and Bayesian coalescent population phylogenies were used as input for the cophylogenetic analyses (described below).

The Bayesian phylogenies also provided the basis for the second test of population monophyly. In this test, hypotheses of population divergence (at the level of country or morph versus species) were tested by comparing the coalescent likelihood [33] and population tree posterior [52] calculated under the Bayesian multi-population coalescent model [33]. The coalescent likelihood calculates the probability of each gene tree *g* given the population tree *S*, as follows:
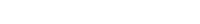


where *b* denotes the branches of the population tree *S*, 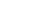 is the implied history of *g* over *b*, and 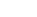 is the function for effective population size through time [33]. The population tree posterior is the sum of that tree's log likelihood and log prior probability, plus the log prior probability densities for any other included priors [52]. For each of these parameters, Tracer was used to calculate the mean value across the MCMC samples (excluding the burn-in) as well as the 95% Bayesian confidence interval (CI), which is the shortest interval containing 95% of the sampled values. The preferred phylogenetic hypothesis, in this test, is the one with the highest coalescent likelihood and tree posterior. Comparing these parameter values between population trees allowed us to evaluate independent phylogenetic estimates for each geographical sampling level (and so did not require nested hypotheses of population monophyly, as does the Bayesian coalescent method for population delimitation of Yang and Rannala [53] for example). The Bayesian coalescent analysis was based on a reduced dataset consisting of those country level populations which were sampled at all four gene loci, according to the requirements of *BEAST. Biogeographic region was not included as a population level in the Bayesian coalescent analyses, due to the unavailability of gene sequences with sufficient coverage of the four included loci.

Population divergence times were estimated in *BEAST under the Bayesian coalescent model [33], which estimates and incorporates both phylogenetic branching patterns and effective population sizes. Such methods, which explicitly model gene lineage coalescence, are expected to give relatively accurate estimates of divergence times compared, for example, to estimates from individual or concatenated gene loci [54]. Two time-calibration methods were used. First, a relaxed, uncorrelated log-normal molecular clock was used to estimate divergence times based on a rate of 0.01909 substitutions per site per million years [35]. This rate was taken from a recently published fossil-calibrated molecular clock estimate among 7 loci (including both mitochondrial and nuclear genes) for a butterfly clade (Papilioninae, Papilionidae) [35] and is very close to Brower's [34] widely used arthropod molecular clock rate for mitochondrial loci of 0.02 substitutions per site per million years [35]. This substitution rate was set for one reference locus (COI) and specified as a prior for the 3 remaining loci (COII, Mpi, Tpi), after Heled and Drummond [33]. This analysis produced tree topologies identical to those of the *BEAST analyses (described above), in which no time-calibrated substitution rate was specified (there, the reference locus rate and priors for the other loci were set to 1, giving branch lengths in units of substitutions per site). A second analysis used a time calibration interval for the divergence of genus *Heliconius* from genus *Eueides*, based on a fossil-calibrated phylogeny of the butterflies [36]. This interval, from 12–23 Mya, was specified as a uniform prior on the age of the phylogenetic root node, based on the published 95% Bayesian confidence interval for the *Heliconius*-*Eueides* divergence time estimated at 18 Mya [36].

### Cophylogenetic analyses

The MDC and Bayesian coalescent population phylogenies of *H. erato* and *H. melpomene* were used to conduct cophylogenetic analyses, using TreeMap 3 [55] and Jane 3 [56]. These analyses tested for statistically significant topological congruence between two given phylogenies (here, corresponding phylogenetic estimates for *H. erato* and *H. melpomene*, as described below), as would be compatible with a history of codivergence between mimetic populations. Cophylogeny mapping reconstructs histories that explain the similarities and differences between associated phylogenies given a cost regime for the recoverable historical events [56], [57]. This is achieved by mapping current ecological associations (e.g. between mimics and models) back into the internal nodes of one phylogeny (e.g. that of the model) to reconstruct a cophylogenetic history (e.g. the history of mimicry between two species). In our context, the recoverable historical events are *codivergence* (parallel divergence of mimetic lineages), *duplication* (divergence of mimic lineages without model divergence), *model switch* (divergence of a mimic lineage onto an additional model lineage), and *loss* (absence of a mimic on a model lineage where it would otherwise be expected).

Müllerian co-mimics may benefit from a shared warning pattern to different degrees [3], [16]. *Heliconius erato* has several characteristics, independent of hypothetical divergence times, which suggest that it has had the dominant role in its mimicry relationship with *H. melpomene* (as suggested by Eltringham in 1916 [16], [58]). These include generally greater current [16], and possibly historical [29], abundance, greater gregariousness, a wider geographic distribution, and pupal mating [16]. Therefore, we treated *H. erato* as the model and *H. melpomene* as the mimic in our main cophylogeny mapping analyses, conducted using TreeMap and Jane. For comparison, these analyses were also repeated with a reversed model-mimic relationship.

Cophylogeny mapping in Jane uses heuristics to find solutions that minimise the overall cost of a historical reconstruction given a cost regime. The default event costs are zero for a codivergence event, one for duplication and model switch events, and two for loss events. TreeMap 3 attempts to find a Pareto set of solutions, that is, all the histories that could be optimal, given the input phylogenies and associations, under a range of event cost regimes. This range is very permissive: codivergence is set at zero cost and all the other costs are assumed to be positive, but do not need to be specified. Statistical analysis can then be performed (in both programs) to test whether the cost of a historical reconstruction is significantly lower than expected by chance, by generating a pseudo-random sample of minimal costs from a null distribution of problem instances with the same model phylogeny. The null distribution is generated by repeatedly randomising either the associations between the leaves (also known as the terminal taxa) or the branching order of the associate (mimic) tree. Thus there are two null hypotheses we might test: either (a) the current associations between model and mimic are not a consequence of a history of coevolution with the model phylogeny, or (b) the branching order of the mimic tree is not dependent on the branching order of the model tree. We prefer the latter test as it accounts for differences in probability of different tree shapes (by randomising the mimic phylogeny), but we conducted both tests for completeness and comparability with other studies.

An additional pairwise distance correlation test of topological congruence was performed in TreeMap 3. This test compares the significance of correlations of pairwise distances between leaves, for associated clades in the two phylogenies, against a distribution of such measures estimated by randomising subtrees of the mimic phylogeny. Each non-trivial internal node of each phylogeny is tested as follows. The leaves descended from the node are determined and their associated leaves in the other phylogeny are found, then the most recent common ancestor of those associated leaves is found. Thus we determine associated subtrees, which are then tested by randomising the mimic subtree. The test statistic is the Spearman correlation of distances between corresponding pairs of leaves. This provides a general test of the topological congruence between two associated phylogenies that does not require an explicit estimate of the history of associations between them. Rather, it tests against a null hypothesis that the mimic phylogeny is independent of the model phylogeny.

To ensure that the results of the cophylogenetic analyses were not exclusive to our coalescent population phylogenies, a cophylogenetic pairwise distance correlation test (as described above) was performed (i) on recent, genome-wide, AFLP phylogenies of *H. erato* (including 85 specimens) and *H. melpomene* (including 78 specimens) [30]; and (ii) on phylogenies reconstructed from a recently published 10-gene dataset (with 127 specimens), which included 5 linked genes involved in heliconian wing colour pattern determination [22]. To produce the input for these cophylogenetic analyses, the topologies of the published AFLP phylogenies [30] were replicated and the 10-gene dataset [22] was downloaded from GenBank (accession numbers from Table S1 in Hines et al. [22]) and re-analysed. For the 10-gene dataset, MUSCLE was used to produce separate gene locus alignments for *H. erato* (plus its relatives *H. himera* and *H. clysonymus*) and *H. melpomene* (plus its relatives *H. cydno*, *H. ismenius* and *H. numata*). Alignments for each gene locus were then concatenated to produce two multilocus data matrices: one including all 10 genes and the other containing only the 5 colour pattern genes. A ML phylogeny was then reconstructed for each multilocus data matrix in Treefinder, using a partitioned nucleotide substitution model with the best-fit substitution model for each gene selected by jModelTest under the AIC. For each of these phylogenies, monophyletic clades of each wing pattern morph were then collapsed to a single leaf, to avoid pseudo-replication of mimicry associations.

## Results

### Phylogenetics and population genetics

Shimodaira-Hasegawa character support tests [41] on monophyly-constrained ML trees could not reject population monophyly at the level of country, biogeographic region, or morph for *H. erato* or *H. melpomene* (*p*>0.4 in all cases). Taking into account the number of parameters imposed by each monophyly constraint, using the AIC [46] difference from the unconstrained ML tree (corresponding to consistent species level monophyly only), *country* was the favoured monophyly level for the sampled within-species populations (AIC scores: ML tree  = 44,033, countries  = 47,500, regions  = 47,780, morphs  = 47,502; AIC difference: countries  = 3,468, regions  = 3,747, morphs  = 3,470). Similarly, the highest coalescent likelihood mean (clm) and population tree posterior (ptp) values were shown by the Bayesian phylogenies of the country level populations, indicating that the Bayesian multi-population coalescent model [33] also favoured population divergence at the level of *country* (corresponding phylogenies Figures S1 and S2) over divergence at the level of *species* or *morph* (corresponding phylogenies Figures S3 and S4): country level clm  = 1,774 [1,685 to 1,862]; ptp  = −17,478 [−17,598 to −17,359]; species level clm  = 1,220 [1,130 to 1,310], ptp  = −18,287 [−18,399 to −18,179]; morph level clm  = 1,701 [1,613 to 1,788], ptp  = −17,617 [−17,732 to −17,503]. The Bayesian CI values for the species level phylogeny do not overlap with those for the other population levels. However, the overlap in 95% confidence intervals for the population levels of country and morph indicates that there is relatively little information by which to choose between these hypotheses.

Monophyly of sampled morph populations (at least at the level of country) is supported by the gene sequence data and provides the most probable coalescent history for the sampled gene loci. This concurs with the greater clustering of individuals into monophyletic country level populations observed on recent genome-wide AFLP phylogenies, relative to phylogenetic estimates based on three concatenated mitochondrial loci [30]. These results (see also [15], [30]) do suggest that the wing pattern morphs sampled from multiple countries (here *H. erato hydara*, *H. erato petiverana*, *H. melpomene melpomene* and *H. melpomene rosina*) may be non-monophyletic. However, neutral markers for recently diverged populations that can experience ongoing low-level gene flow (including those used in this study) may show relatively low levels of phylogenetic structure [22] and we note that the character support analyses were unable to reject monophyly of the higher population levels of biogeographic region or morph. Based on the character support and Bayesian coalescent analyses, we therefore focussed our cophylogenetic analyses on the country level populations, which received the highest monophyly support. However, phylogenies for region and morph level populations, which received lesser support, were also analysed.

### Topological congruence between the population phylogenies of *H. erato* and *H. melpomene*

**Figure 2:**
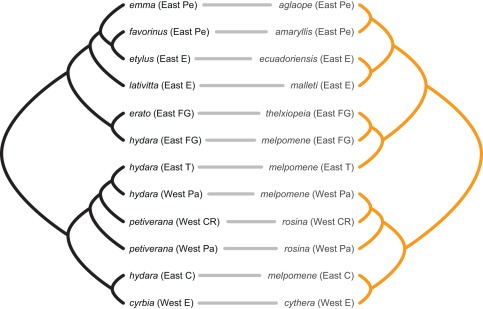
Phylogenies of *H. erato* and *H. melpomene* illustrating branching orders of co-mimetic country level populations within each species. Example phylogenies independently estimated for *H. erato* (black, left) and *H. melpomene* (orange, right) using the Minimise Deep Coalescence (MDC) method [32]. These correspond to cophylogenetic analysis “separate MDC countries 1” in Table 1. *H. erato*/*H. melpomene* co-mimics (see Figure 1) are indicated by grey lines. This is one of several possible phylogeny pairs with similarly high congruence (see Table 1). Taxon labels indicate the sampled biogeographic region (East or West of the Andes), and country (abbreviations are: CR Costa Rica, Pa Panama, E Ecuador, C Colombia, FG French Guiana, T Trinidad and Pe Peru).

**Figure 3:**
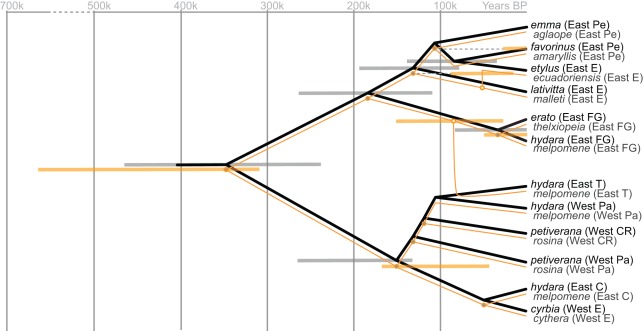
Cophylogenetic reconstruction of the history of mimicry between country level populations of *H. erato* and *H. melpomene*. Example cophylogenetic history of the mimicry relationship between *H. erato* and *H. melpomene* based on MDC phylogenetic estimates (shown in Figure 2), and reconstructed using TreeMap 3. Bars indicate 95% Bayesian confidence intervals for divergence times, corresponding to Figure S1. Grey-filled dots correspond to reconstructed codivergence events; white-filled dots represent duplication events which, in this case, are both followed by model switch events – one for the colonisation of *H. erato etylus* in East Ecuador by *H. melpomene ecuadoriensis*, here the sister to *H. melpomene malleti* (also from East Ecuador), the other for the colonisation of the *H. erato hydara* population from Trinidad by a population of *H. melpomene melpomene*, here the sister of a lineage from French Guiana (one of the sampled mainland countries closest to Trinidad); the only mimicry loss event is indicated at the most recent common ancestor of *H. erato hydara* populations from Trinidad and Panama. Taxon labels correspond to those in Figure 2.

To account for phylogenetic uncertainty, we conducted cophylogenetic analyses across the set of phylogenies returned by the coalescent analyses (listed in Table 1). Across these MDC and Bayesian coalescent population phylogenies, the overwhelming indication is of significant topological congruence (Table 1). The cophylogenetic analyses suggest that, in almost all cases, there are more codivergence events between the mimetic populations of *H. erato* and *H. melpomene* than would be expected by chance if their phylogenies were independent. Figure 2 shows an example pair of phylogenies from this set of phylogenetic estimates with similarly high congruence (see Table 1). Reconciling the phylogeny of *H. melpomene* with that of *H. erato* indicates remarkable topological congruence, with 8 codivergence events out of out of a possible 11, two duplications followed by model switches, and one loss (Figure 3). Interestingly, we still obtain highly significant congruence between the phylogenies when the mimic-model relationship is reversed (Table S2).

The pairwise distance correlation test also showed significant congruence at the roots of the *H. erato* and *H. melpomene* phylogenies, for the majority of our phylogenetic estimates (Table 1), suggesting that the phylogenies have been highly dependent on each other throughout their history.

Pairwise distance correlation tests conducted on the recent AFLP phylogenies [30] (Figure S5) and on the 10-gene dataset phylogenies [22] (Figures S6 and S7) also suggest significant topological congruence between the radiations of *H. erato* and *H. melpomene* (Table 1), contrary to the conclusions of these authors [22], [30].

### Temporal congruence between the population phylogenies of *H. erato* and *H. melpomene*

Using the program SITES [47], we estimated average uncorrected genetic divergence (average pairwise substitutions per site, excluding gaps and indels) at 0.0245 within *H. erato* and 0.0153 within *H. melpomene* (excluding other, putatively incipient, species). Thus, the average uncorrected sequence divergence among individuals sampled from *H. melpomene* is considerably lower than (62% of) that estimated for *H. erato*, as previously suggested [15]. However, the effective population size parameter for *H. melpomene*, measured as the product of effective population size and mutation rate (θ = 4 N_e_μ), was estimated at 41% to 63% of that for *H. erato*, see also [29]. Specifically, the value of θ calculated using the method of Watterson [48] was 0.1058 for *H. erato* and 0.0429 for *H. melpomene*, and the θ value calculated based on pairwise nucleotide diversity (see [47]) was 0.0245 for *H. erato* and 0.0153 for *H. melpomene*, with each estimate of θ calculated as an average per base pair, with gaps, indels and sequences for putatively incipient species excluded [47]. Effective population size is known to be positively correlated with average genetic diversity. Therefore, the lower average pairwise genetic diversity of *H. melpomene*, relative to *H. erato*, is an expected consequence of a lower effective populations size (e.g. see [49]), and is compatible with similar origination dates for the sampled clades of *H. melpomene* and *H. erato*, as discussed below.

In results similar to those of Flanagan et al. [29], average uncorrected sequence divergence between *H. erato* and the closely related species *H. hecalesia* (0.0605) was greater than that between *H. melpomene* and its close relative *H. cydno* (0.0303), or that between *H. melpomene* and the silvaniforms (0.0512), which form the outgroup to *H. melpomene* plus the *H. cydno* group [20]. Divergence times estimated on our Bayesian coalescent phylogenies of the *Heliconius* (Figures S1, S2, S3 S4), using both substitution rate [34], [35] and node age calibrations [36], also suggest that the split between *H. hecalesia* and *H. erato* (country population phylogenies: rate calibrated age 2.8 Mya [1.9, 3.76], node calibrated age 4.57 Mya [2.89, 6.3]; morph phylogenies: rate calibrated age 2.77 Mya [1.82, 3.79], node calibrated age 2.88 Mya [1.41, 4.63]) is older than that between the *H. cydno* species group and its *H. melpomene* sister clade (country populations: rate calibrated age 0.35 Mya [0.23, 0.48], node calibrated age 0.6 Mya; morphs: rate calibrated age 0.4 Mya [0.23, 0.58], node calibrated age 0.43 Mya [0.17, 0.73]), as previously suggested [29], as well as that between *H. melpomene* – plus the *H. cydno* group – and the silvaniforms (country populations: rate calibrated age 1.93 Mya [1.29, 2.64], node calibrated age 2.46 Mya; morphs: rate calibrated age 1.9 Mya [1.29, 2.6], node calibrated age 1.66 Mya [0.8, 2.63]). However, like the population genetic results discussed above, the Bayesian divergence estimates suggest temporal congruence between the population radiations of *H. erato* and *H. melpomene* (Figure 3), contrary to [29]. First, sampled *H. erato* and *H. melpomene* were estimated to have begun their radiations at similar times. Specifically, the age of the most recent common ancestor of the included populations was estimated at 200,000 to 500,000 years for *H. erato* and 200,000 to 800,000 years for *H. melpomene* (*H. erato*: country populations, rate calibrated age 0.35 Mya [0.24, 0.47], node calibrated age 0.49 Mya [0.3, 0.7], morphs, rate calibrated age 0.25 Mya [0.2, 0.34], node calibrated age 0.22 Mya [0.11, 0.36]; *H. melpomene*: country populations, rate calibrated age 0.47 Mya [0.31, 0.66], node calibrated age 0.75 Mya [0.43, 1.11], morphs, rate calibrated age 0.2 Mya [0.11, 0.32], node calibrated age 0.21 Mya [0.08, 0.36]). Second, estimated ages of mimetic populations of *H. erato* and *H. melpomene* were often similar. Of the codivergence events reconstructed for the MDC phylogenies of Figure 3, for example, 95% Bayesian confidence intervals overlap where they are available. A historical reconstruction incorporating the estimated divergence times on the Bayesian country level phylogeny finds 5 codivergence events out of a maximum of 8 (Figure S8). Ages for the first divergence of eastern and western populations of *H. erato* and *H. melpomene* also show overlapping confidence intervals, although the point estimate for *H. erato* was estimated at approximately twice that for *H. melpomene*, as discussed below (*H. erato*: country populations, rate calibrated age 0.2 Mya [0.13, 0.27], node calibrated age 0.32 Mya [0.2, 0.45], morphs, rate calibrated age 0.18 Mya [0.13, 0.24], node calibrated age 0.16 Mya [0.07, 0.25]; *H. melpomene*: country populations, rate calibrated age 0.1 Mya [0.04, 0.17], node calibrated age 0.13 Mya [0.04, 0.23], morphs, rate calibrated age 0.1 Mya [0.05, 0.16], node calibrated age 0.11 [0.04, 0.19]).

## Discussion

### Evidence for codivergence

Our coalescent population phylogenies for *H. erato* and *H. melpomene* (e.g. Figure 2; Figures S1, S2, S3 S4) have many features in common with previous phylogenetic estimates [15], [30], including a strong signal from biogeographic region (East or West of the Andes). The MDC coalescent phylogenies represented in Figure 2, for example, share major topological features with recent, genome-wide, AFLP phylogenies of *H. erato* and *H. melpomene* [30] (Figure S5). These features include a relatively basal split, within each species, between two clades; one clade containing eastern and western populations of, mimetic, *H. erato hydara/H. melpomene melpomene* plus the populations of the other western morphs (mimetic, *H. erato petiverana/H. melpomene rosina* and *H. erato cyrbia/H. melpomene cythera*), the other clade containing the remaining eastern populations of *H. erato hydara/H. melpomene melpomene* plus populations of the other eastern mimetic morphs. Within the solely eastern clades, a basal split between two major sub-clades is also shared with the recent AFLP phylogenies; one sub-clade containing *H. erato hydara*/*H. melpomene melpomene* and the French Guianan population of, mimetic, *H. erato erato*/*H. melpomene thelxiopeia*, the other sub-clade containing populations of, mimetic: *H. erato lativitta*/*H. melpomene malleti*, *H. erato emma*/*H. melpomene aglaope*, *H. erato etylus*/*H. melpomene ecuadoriensis* and *H. erato favorinus*/*H. melpomene amaryllis*.

Cophylogenetic analyses, conducted across the set of coalescent phylogenetic reconstructions, give an overall picture of statistically significant topological congruence between the evolutionary radiations of *H. erato* and *H. melpomene* co-mimics (contrary to previous suggestions [15], [30]) (Table 1). In particular, all phylogenetic estimates for the, best supported, country level populations are compatible with a history of repeated codivergence between mimetic populations.

In the interpretation of their AFLP phylogenies, Quek et al. [30] emphasised elements of incongruence between the topologies for *H. erato* and *H. melpomene*. They [30] noted, specifically, that the earliest branching lineages within each species did not represent co-mimetic morphs (these were *H. erato etylus* sampled from East Ecuador, which instead falls within the eastern clade of our figure 2, and *H. melpomene nanna* sampled from Brazil, which was not included in our coalescent analyses).

However, a cophylogenetic analysis conducted on these recent AFLP phylogenies [30] also indicates that patterns of evolutionary branching among co-mimics are significantly more similar than expected by chance (Table 1; Figure S5), despite elements of incongruence such as those described above. This suggests that an early lack of phylogenetic resolution [15] and as well as the complexity of more recent estimates of phylogenetic branching patterns [30] have previously concealed significant topological congruence between the phylogenies of *H. erato* and *H. melpomene*, which is revealed by quantitative cophylogenetic analysis.

Phylogenies of *H. erato* and *H. melpomene* based on a reanalysis of the complete 10-gene dataset of Hines et al. [22] also show significant topological congruence (Table 1; Figure S6), and biogeographic clustering patterns complementary to those of the coalescent population phylogenies illustrated in Figures 2. Similar reanalyses of only the 5 colour pattern genes from this dataset [22] are less able to cluster individuals of the same morph and show reduced biogeographic signal but also indicate statistically significant topological congruence between phylogenies of *H. erato* and *H. melpomene* co-mimics (Table 1; Figure S7).

As expected for recent evolutionary radiations of populations that still experience low-level gene flow, phylogenetic reconstructions for *H. erato* and *H. melpomene* are subject to some uncertainty, and there are differences between phylogenetic estimates based on different gene partitions (e.g. Figures S6 and S7), taxon partitions, and methodologies (e.g. the MDC and Bayesian phylogenetic estimates shown in Figures 2 and S1, respectively). However, several topological features are common to phylogenetic estimates based on different methodologies and data partitions (as discussed above) and the consistent result that emerges when we consider these various phylogenies is one of statistically significant topological congruence in the branching patterns of co-mimetic populations within these two species.

To be compatible with codivergence, ecologically associated phylogenies must be both topologically and temporally congruent [55]. For example, the phylogenies of *H. erato* and *H. melpomene* might show topological but not temporal congruence if wing patterns arising from an earlier radiation (previously suggested to be that of *H. erato* [15], [29]) were secondarily converged upon during a later, but topologically similar, radiation by a mimic (previously suggested to be *H. melpomene*) [16]. Previous studies have generally suggested that the phylogenies of *H. erato* and *H. melpomene* were temporally incongruent. In 1996 [15], Brower estimated that two eastern clades within *H. erato* and *H. melpomene* were of similar ages (150,000 – 200,000 years old), based on uncorrected average within-clade genetic divergence. However, his estimation that a key divergence between populations East and West of the Andes occurred earlier in *H. erato* (1.5–2 Mya) than in *H. melpomene* (65,000 years ago) [15] has been taken as evidence against codivergence of the two species [15], [27]. In the same vein, Flanagan et al. [29] suggested that *H. erato* was approximately twice as old as *H. melpomene*, based on corrected genetic divergences from their nearest relatives (thought to be *Heliconius hecalesia* and *Heliconius cydno*, respectively). These apparent discrepancies in divergence times have previously been taken as evidence against coevolution of *H. erato* and *H. melpomene* [15], [27].

As in previous studies [15], we find that *H. melpomene* shows lower genetic diversity than *H. erato*, as measured by the average uncorrected pairwise divergence between individuals. However, population genetic comparisons indicate that the overall effective population size (estimated as θ, the product of effective population size and mutation rate) of *H. melpomene* is smaller than that of *H. erato* (observed here and also by Flanagan et al. [29]). The effective population size is the size of an idealized breeding population that would experience the same effects of random mutation as a real population under study [59]. Effective population size is generally positively related to, but less than, the census population size [60]. Therefore field observations suggesting that *H. melpomene* generally has a census population size approximately half that of *H. erato* (e.g. see [16]) are compatible with the difference in effective population size estimated from sampled genetic variation. Within-species genetic diversity is positively correlated with effective population size [49], [60], [61], [62]. Indeed, the population size parameter θ determines the average genetic diversity of the population, because it takes into account both effective population size and mutation rate (these parameters can be separated using independent estimates of the mutation rate, for example from fossil calibrated divergence times [61], however such information is not available for *H. erato* or *H. melpomene*). The estimated difference in effective population size predicts that average genetic variation could be lower within the less abundant species *H. melpomene*, even if its radiation was temporally congruent with that of *H. erato* (e.g. see [49]).

Like Flanagan et al. [29], we estimated splits between *H. melpomene* and closely related clades (the *H. cydno* clade or the silvaniforms) to be younger than the split between *H. erato* and its close relative *H. hecalesia* (Figures S1, S2, S3 S4). This finding was supported both by differences in average sequence divergence (here and also Flanagan et al. [29]) and by the Bayesian coalescent phylogenetic analyses (which are more robust, since they estimate and take into account effective population sizes [33]). However, the crucial test for codivergence is not whether *H. erato* and *H. melpomene* first diverged from respective outgroups at similar times but whether their internal population radiations were temporally congruent.

Our Bayesian coalescent reconstructions indicate similar ages for the most recent common ancestor of the sampled populations of *H. erato* (200,000 to 500,000 years) and *H. melpomene* (200,000 to 800,000 years), with overlapping 95% Bayesian confidence intervals (e.g. Figure 3). This is compatible with a contemporaneous codivergence event at the start of the sampled radiations of these species (e.g. Figure 3). Thus we concur with Brower's [15] hypothesis that much of the phenotypic diversity within *H. erato* and *H. melpomene* evolved relatively recently, but estimate the origin of the sampled morphs of *H. erato* to be more recent than his estimate of 1.5–2 MY, and contemporaneous with that of *H. melpomene*, contrary to his conclusions [15]. The age of the first divergence between eastern and western populations (see Figure 3; and Figures S1, S2, S3 S4) was estimated at 160,000 to 320,000 years for *H. erato* and at 100,000 to 130,000 years for *H. melpomene* (though a smaller sample of 2 western populations were included in the Bayesian analyses for *H. melpomene*, compared to 3 western populations of *H. erato*). However, the 95% Bayesian confidence intervals overlap, suggesting that contemporaneous codivergence of western and eastern populations, within the two species, cannot be rejected, contrary to previous suggestions [15].

Overall, the population genetic and Bayesian coalescent divergence time estimates strongly suggest that the parallel phenotypic radiations of *H. erato* and *H. melpomene* occurred during an overlapping time period, contrary to previous suggestions [15], [29], [30]. The phylogenetic reconstructions and divergence time estimates are compatible with a series of contemporaneous codivergence events, occurring during a Müllerian mimicry relationship sustained over at least 350,000 years.

### Codivergence and coevolution

Congruent phylogenies are often considered necessary to sustain hypotheses of (strictly reciprocal) coevolution [11], [15]. Here, we find significant topological and temporal congruence between the phylogenies of *H. erato* and *H. melpomene*, which demonstrates that coevolution between the two species was possible (contrary to some previous suggestions [15], [30]). Furthermore, codivergence can be considered some of the strongest evidence that coevolution did occur [25], [26]. In the case of *H. erato* and *H. melpomene*, the codivergent populations identified by the cophylogenetic reconstructions frequently represent distinct mimetic wing patterns (e.g. Figure 1). Thus, population codivergence is correlated with parallel genetic [3], [63] and phenotypic [3] variation. When sustained codivergence is accompanied by multiple examples of parallel phenotypic change (as in the co-mimetic morphs of *H. erato* and *H. melpomene* illustrated in Figure 1), reciprocal coevolution can be considered a more probable mechanism than, for example, secondary, one-sided evolutionary change by one species (previously suggested to be the less abundant *H. melpomene*) to match its co-mimic (previously suggested to be the more abundant *H. erato*) [14], as follows.

As noted previously [14], evidence for topological and temporal congruence between associated phylogenies, such as that presented here, cannot completely reject the possibility that one of the taxa in question (such as *H. erato*) radiated first (within the limits of the overlapping Bayesian confidence intervals) and prompted a second parallel radiation by the other taxon (such as *H. melpomene*). It has been suggested to be unlikely that, in every case, mutations associated with parallel wing pattern change would occur first in *H. erato* and second in *H. melpomene* over multiple codivergence events [14]. A generally larger population size (as estimated here and suggested previously [16], [29]) may have made such wing pattern mutation in *H. erato* more probable (e.g. [64]). However, some parallel wing pattern changes between the co-mimics may have been prompted by an initial mutation in *H. melpomene*, although uncertainty in divergence time estimates make the precise order of divergences extremely difficult to determine.

Perhaps more relevant to the argument for coevolution, *sensu stricto*, of *H. erato* and *H. melpomene* [12], [13] is whether their evolution was reciprocal, in that change in one species exerted a selection pressure on the other and vice versa. Müllerian mimicry theory [6] predicts that two unpalatable co-mimics will both benefit from a shared warning pattern, although a less abundant species will receive greater fitness gains than a more abundant co-mimic (as introduced above). For example, based on our estimate that the effective population size for *H. erato* is, overall, approximately twice that for *H. melpomene*, the classical Müllerian model would predict mutual fitness benefits with a, respective, ratio of 1∶4 (e.g. see [11]). While this supports previous suggestions that *H. erato* has generally played the dominant role in its mimicry relationship with *H. melpomene* (e.g. [16]), relative abundances at approximately these levels have been predicted to promote reciprocal coevolution involving convergent evolutionary change (e.g. see Figure 2 of [14] and Figure 7 of [65]). Furthermore, field studies demonstrate that *Heliconius* abundance can fluctuate both regionally and seasonally, and the local population size of *H. melpomene* can equal or exceed that of *H. erato* [66]. Potentially this would create mutual peaks in selection for Müllerian mimicry during periods of similar abundance, for example at shared population bottlenecks [66]. Codivergence may not prove coevolution in the strict sense [14]. However, where there is a history of codivergence [25], such as that suggested by this study – particularly between ecological associates predicted to exert reciprocal selection pressures, such as these Müllerian co-mimics – at least some degree of coevolution is strongly suggested. Biogeographic comparisons suggesting that the dominant model *H. erato* has sometimes converged towards *H. melpomene* [14] offer another compatible line of evidence for reciprocal evolutionary change. While theory suggests that the mutual fitness benefits of Müllerian mimicry will promote coevolution [3], [6], [11], evidence for this has previously been rare [16]. Therefore, our evidence for sustained codivergence between mimetic populations of *H. erato* and *H. melpomene* represents an important empirical case for the study of coevolution.

Coevolution is a powerful concept because it describes a mechanism for the coordination of evolutionary change in genetically separate populations [13]. Consequently, evidence for coevolution has fundamental implications for ecology, population genetics and wider evolutionary theory [13]. Here, we have presented evidence for phylogenetic codivergence between mimetic populations of *H. erato* and *H. melpomene*. Such codivergence represents some of the strongest evidence for coevolution [14], [25]. Therefore, the parallel radiations of *H. erato* and *H. melpomene* support a hypothesis of reciprocal coevolution between Müllerian co-mimics characterised by population codivergence and parallel phenotypic change [3]. Consequently, we suggest that these parallel radiations deserve to be reinstated (after [14], [67]) as one of the most striking known examples of coevolution.

## Supporting Information

Figure S1 Bayesian coalescent phylogeny for the *Heliconius* with country level populations of *H. erato* and *H. melpomene*. Branch labels give posterior probabilities, the axis indicates time (Mya) based on substitution rate calibration, and scale bars show 95% Bayesian confidence intervals for the mean node age.
(EPS)

Figure S2 Bayesian coalescent phylogeny for the *Heliconius* with country level populations of *H. erato* and *H. melpomene*, labelled as for Figure S1 and showing a time axis based on node age calibration.
(EPS)

Figure S3 Bayesian coalescent phylogeny for the *Heliconius* with morph level populations of *H. erato* and *H. melpomene*, labelled as for Figure S1 and showing a time axis based on substitution rate calibration.
(EPS)

Figure S4 Bayesian coalescent phylogeny for the *Heliconius* with morph level populations of *H. erato* and *H. melpomene*, labelled as for Figure S1 and showing a time axis based on node age calibration.
(EPS)

Figure S5 Phylogenies for *H. erato* (left) and *H. melpomene* (right) reproduced from [30] and corresponding to cophylogenetic analysis “Quek et al., 2010 AFLP” in Table 1. *H. erato*/*H. melpomene* co-mimics sampled from the same country are indicated by grey lines. Taxon labels indicate the sampled biogeographic region (East or West of the Andes), and country (abbreviations correspond to Figure 2). Shaded circles indicate the significance of a pairwise distance correlation test conducted for the shaded node (with *p* values corresponding to the key).
(EPS)

Figure S6 Maximum likelihood phylogenies independently estimated for *H. erato* (left) and *H. melpomene* (right) based on the 10-gene dataset of [22], corresponding to cophylogenetic analysis “Hines et al. 2011, 10-genes” in Table 1. Taxon labels indicate the sampled biogeographic region (abbreviations are: Am Amazon, Ca Caribbean, Ch Chocoan-Parana), and country (abbreviations correspond to Figure 2 with additional abbreviation: B Brazil). Further annotation corresponds to Figure S5.
(EPS)

Figure S7 Phylogenies reconstructed as for those of Figure S6 except based on only the 5 colour pattern genes of [22] and corresponding to cophylogenetic analysis “Hines et al. 2011 colour pattern genes” in Table 1. Further annotation corresponds to Figure S5.
(EPS)

Figure S8 History of mimicry between *H. erato* (black phylogeny) and *H. melpomene* (blue phylogeny), reconstructed using Jane 3, based on the phylogeny shown in Figure S1: white-filled circles represent codivergence, solid circles represent duplications, arrows represent model switches, and dashed lines represent losses.
(EPS)

Table S1 Sampling information for the study system, including accession numbers.
(XLS)

Table S2 Significance of congruence between phylogenies with a model (here *H. melpomene*) to mimic (here *H. erato*) relationship, reversed relative to Table 1.
(DOC)

Table S3 DNA sequences used in this study: aligned data matrix in Nexus format.
(TXT)
