# Supplementary material for: Phylogenetic Codivergence Supports Coevolution of Mimetic Heliconius Butterflies
Source: PLoS One. 2012 May 7;7(5):e36464. doi: 10.1371/journal.pone.0036464 (PMC3346731; doi:10.1371/journal.pone.0036464)
Supplement: Table S2 — Significance of congruence between phylogenies with a model (here H. melpomene) to mimic (here H. erato) relationship, reversed relative to Table 1. (DOC) [file pone.0036464.s010.doc]

| **Phylogenies** | **Minimum Cost**  **(p [95% max])** | | **Distance Correlation**  **(p [95% max])** | |
| --- | --- | --- | --- | --- |
|  | Random Associations | Random Mimic Tree | Root of Mimic  (*H. erato*) | Root of Model  (*H. melpomene*) |
| separate MDC countries 1 | 0 [0.0024] | 0 [0.0024] | 0.002 [0.0059] | 0.001 [0.0042] |
| separate MDC countries 2 | 0 [0.0024] | 0 [0.0024] | 0 [0.0024] | 0 [0.0024] |
| separate MDC regions 1 | 0.002 [0.0059] | 0.007 [0.0130] | 0 [0.0024] | 0 [0.0024] |
| separate MDC morphs 1 | 0.018 [0.0270] | 0.017 [0.0257] | 0.002 [0.0059] | 0.001 [0.0042] |
| combined MDC countries 1 | 0.007 [0.0103] | 0.007 [0.0130] | 0 [0.0024] | 0 [0.0024] |
| combined MDC countries 2 | 0.001 [0.0042] | 0.003 [0.0074] | 0 [0.0024] | 0.001 [0.0042] |
| combined MDC countries 3 | 0.004 [0.0089] | 0.004 [0.0089] | 0 [0.0024] | 0 [0.0024] |
| combined MDC countries 4 | 0.005 [0.0103] | 0.004 [0.0089] | 0 [0.0024] | 0.003 [0.0074] |
| combined MDC regions 1 | 0.123 [0.1439] | 0.121 [0.1418] | 0.01 [0.0170] | 0.01 [0.0170] |
| combined MDC morphs 1 | 0.665 [0.6948] | 0.591 [0.6220] | 0.07 [0.0864] | 0.062 [0.0776] |
| combined *BEAST countries | 0.009[0.0157] | 0.009 [0.0157] | 0.096 [0.1148] | 0.095 [0.1138] |
| combined *BEAST morphs | 0.012 [0.0195] | 0.007 [0.0130] | 0.01 [0.0170] | 0.009 [0.0157] |
